# Association between Protein-Bound Uremic Toxins and Asymptomatic Cardiac Dysfunction in Patients with Chronic Kidney Disease

**DOI:** 10.3390/toxins10120520

**Published:** 2018-12-05

**Authors:** Shanmugakumar Chinnappa, Yu-Kang Tu, Yi Chun Yeh, Griet Glorieux, Raymond Vanholder, Andrew Mooney

**Affiliations:** 1 Department of Nephrology, Doncaster and Bassetlaw Teaching Hospitals, Doncaster DN2 5LT, UK; 2 Leeds Institute of Cardiovascular and Metabolic Medicine, University of Leeds, Leeds LS2 9DA, UK; andrew.mooney2@nhs.net; 3 Institute of Epidemiology & Preventive Medicine, College of Public Health, National University of Taiwan, Taipei 100, Taiwan; yukangtu@ntu.edu.tw (Y.-K.T.); yehyichun@cph.ntu.edu.tw (Y.C.Y.); 4 Department of Nephrology, Ghent University Hospital, 9000 Ghent, Belgium; griet.glorieux@ugent.be (G.G.); raymond.vanholder@ugent.be (R.V.); 5 Department of Nephrology, Leeds Teaching Hospitals NHS Trust, Leeds LS9 7TF, UK

**Keywords:** uremic toxins, cardiac function, cardiac power, aerobic exercise capacity

## Abstract

Although the relationship between protein-bound uremic toxins (PBUTs) and cardiac structure and cardiac mortality in chronic kidney disease (CKD) has been studied in the past, the association between cardiac dysfunction and PBUTs has not yet been studied. We therefore evaluated the association between impaired peak cardiac performance and the serum free and total concentrations of potentially cardiotoxic PBUTs. In a cross-sectional study of 56 male CKD patients (stages 2–5 (pre-dialysis)) who were asymptomatic with no known cardiac diseases or diabetes we measured peak cardiac power (CPO_max_), aerobic exercise capacity (VO_2max_), and echocardiographic parameters of cardiac morphology and evaluated their association with PBUTs. The serum total and free concentrations of indoxyl sulfate (IXS), p-cresyl sulfate (PCS), p-cresyl glucuronide, indole acetic acid, and hippuric acid showed significant negative correlation with CPO_max_ and VO_2max_. IXS and PCS were independently associated with CPO_max_ and VO_2max_ even after controlling for eGFR. No correlation between left ventricular mass index (LVMI) and PBUTs was seen. The present study for the first time has demonstrated the association between subclinical cardiac dysfunction in CKD and serum levels of a panel of PBUTs. Further studies are required to evaluate the mechanism of cardiotoxicity of the individual uremic toxins.

## 1. Introduction

Cardiovascular disease (CVD) is the predominant cause of mortality and morbidity in chronic kidney disease (CKD) and end-stage renal disease (ESRD). Several pathophysiological mechanisms are being studied to understand the mechanism of CVD in CKD. Among the potentially putative agents of CVD in CKD, there is a growing interest in the role of protein-bound uremic toxins (PBUTs) [1,2]. PBUTs such as indoxyl sulfate (IXS) and p-cresyl sulfate (PCS) have been shown to elicit a wide range of toxicity such as endothelial dysfunction, vascular calcification, and induction of oxidative stress [3,4,5,6,7]. More importantly, clinical studies have shown that some of the PBUTs are associated with cardiovascular and all-cause mortality [5,8]. However, the association between cardiac dysfunction and PBUTs has not yet been studied.

In our recently published paper we demonstrated the presence of asymptomatic cardiac dysfunction in CKD patients (stages 2–5 (pre-dialysis)) with no pre-existing cardiac disease or diabetes mellitus [9]. The study also evaluated the association between asymptomatic cardiac dysfunction in CKD and the conventional renal biochemical parameters such as serum creatinine, urea, inorganic phosphate, calcium, and bicarbonate. In the present study, we evaluated the association between such subclinical cardiac dysfunction and the serum levels of a panel of potentially cardiotoxic PBUTs. In addition, we also evaluated the association between PBUTs and cardiac dimensions and aerobic exercise capacity.

## 2. Results

### 2.1. Patient Characteristics

There were 56 male CKD patients with a mean age of 46.8 ± 12.5 years covering the spectrum of CKD from stages 2 to 5 (pre-dialysis). The mean eGFR was 38.5 ± 24.1 mL/min/1.73 m^2^. The patient characteristics such as anthropometry, biochemistry, and parameters of central haemodynamics and exercise capacity across the CKD stages are presented in Table 1. The etiologies of CKD were as follows (number of patients in brackets): IgA nephropathy (16), polycystic kidney disease (12), reflux nephropathy and chronic pyelonephritis (13), membranoproliferative glomerulonephritis (3), Alport’s nephropathy (2), hypertensive nephropathy (1), focal segmental glomerulosclerosis (2), minimal change disease (1), and uncertain etiology (6). None of the study participants had any history of ischaemic, arrhythmic, or valvular heart diseases or diabetes mellitus. Patients were asymptomatic from a cardiac point of view (all NYHA class I). No patient had angina or ECG evidence of cardiac ischaemia or arrhythmia during cardiopulmonary exercise testing (CPX). Of the 56 patients, 62.5% were receiving angiotensin-converting enzyme inhibitors (ACE-I), 34.5% angiotensin receptor blockers, 19.6% β-blockers, and 25% statins.

**Figure 1:**
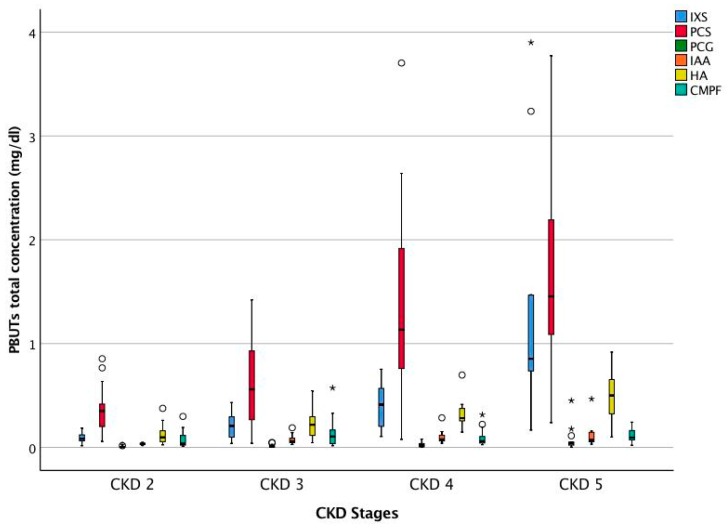
Serum total concentration of PBUTs across CKD stages. CKD 2 (*n* = 14), CKD 3 (*n* = 15), CKD 4 (*n* = 16), and CKD 5 (*n* = 11). Outliers: ° >1.5 × interquartile range (IQR) and * >3.0 × IQR. Indoxyl sulfate (IXS), p-cresyl sulfate (PCS), p-cresyl glucuronide (PCG), indole acetic acid (IAA), Hippuric acid (HA), and 3-carboxy-4-methyl-5-propyl-2-furanpropionic acid (CMPF).

**Figure 2:**
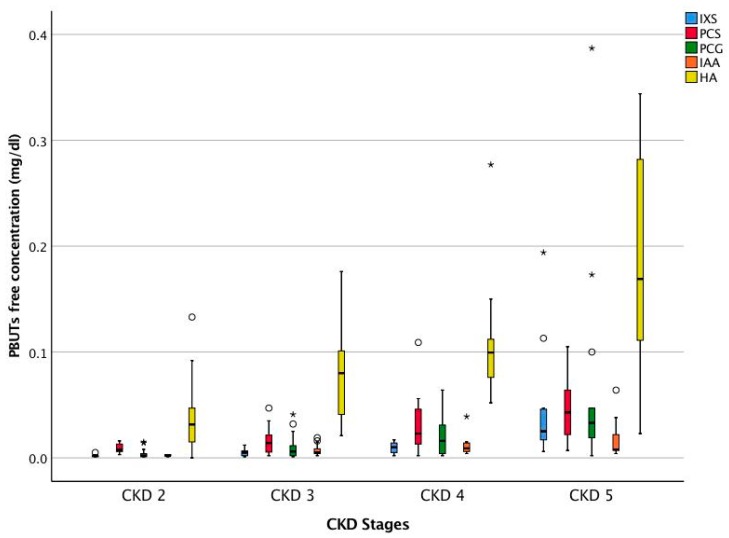
Serum free concentration of PBUTs across CKD stages. CKD 2 (*n* = 14), CKD 3 (*n* = 15), CKD 4 (*n* = 16), and CKD 5 (*n* = 11). Outliers: ° >1.5 × IQR and * >3.0 × IQR. Indoxyl sulfate (IXS), p-cresyl sulfate (PCS), p-cresyl glucuronide (PCG), indole acetic acid (IAA), and Hippuric acid (HA).

The free and total concentrations of the PBUTs such as Indoxyl sulfate (IXS), p-cresyl sulfate (PCS), p-cresyl glucuronide (PCG), indole acetic acid (IAA), hippuric acid (HA), and 3-carboxy-4-methyl-5-propyl-2-furanpropionic acid (CMPF) of the full CKD cohort are presented in Table 2. Figure 1 and Figure 2 show the total and free concentrations of the PBUTs across CKD stages. Since CMPF is bound to protein for nearly 100%, only total concentrations are reported. The difference in the free concentrations of all PBUTs across the CKD stages was significant on ANOVA (all *p* < 0.05). On multiple comparisons, for all PBUTs, the free concentration in CKD 5 was significantly higher compared to those in CKD 2 and 3. For IXS and PCG, the free concentration in CKD 5 was significantly higher compared to those in CKD stages 2, 3, and 4.

### 2.2. Association between PBUTs and Peak Cardiac Performance

#### 2.2.1. Peak Cardiac Power Output (CPO_max_)

The total and free concentrations of IXS, PCS, PCG, IAA, and HA showed significant negative correlation with CPO_max_. No significant correlation existed between CMPF and CPO_max._ (Table 3 and Table 4).

#### 2.2.2. Peak Cardiac Output (Peak Q_t_)

The total and free concentrations of IXS, PCS, PCG, IAA, and HA showed significant negative correlation with peak Q_t_, the volume generating capacity of the heart. No correlation was seen with CMPF (Table 3 and Table 4).

#### 2.2.3. Peak Mean Arterial Pressure (Peak MAP)

The total and free concentrations of PCS and PCG showed significant negative correlation with peak MAP, the pressure generating capacity of the heart. No correlation was seen between IXS, IAA, HA, or CMPF and peak MAP (Table 3 and Table 4).

#### 2.2.4. Peak Heart Rate (Peak HR)

The total concentrations of PCG, IAA, and HA and the free concentrations of PCG and IAA showed significant negative correlation with the peak HR. The other toxins did not show any significant correlations with peak HR (Table 3 and Table 4). 

### 2.3. Association between PBUTs and Aerobic Exercise Capacity

#### 2.3.1. Aerobic Exercise Capacity (VO_2max_)

The total and free concentrations of IXS, PCS, PCG, IAA, and HA showed significant negative correlation with VO_2max_. No significant correlation existed between CMPF and VO_2max_ (Table 3 and Table 4).

#### 2.3.2. Arteriovenous O_2_ Difference [C(a-v)O_2_]

The total concentrations of PCS, PCG, and IAA and free concentrations of PCS and IAA showed significant negative correlation with C(a-v)O_2_ (Table 3 and Table 4).

### 2.4. Independent Association of PBUTs with CPO_max_ and VO_2max_

**Figure 3:**
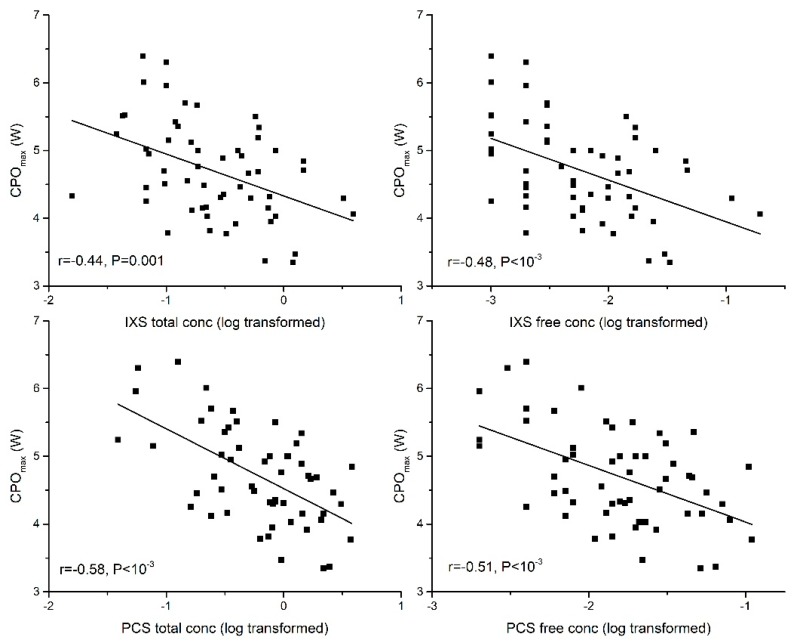
Association of serum total and free concentrations of indoxyl sulfate (IXS) and p-cresyl sulfate (PCS) with peak cardiac power (CPO_max_).

**Figure 4:**
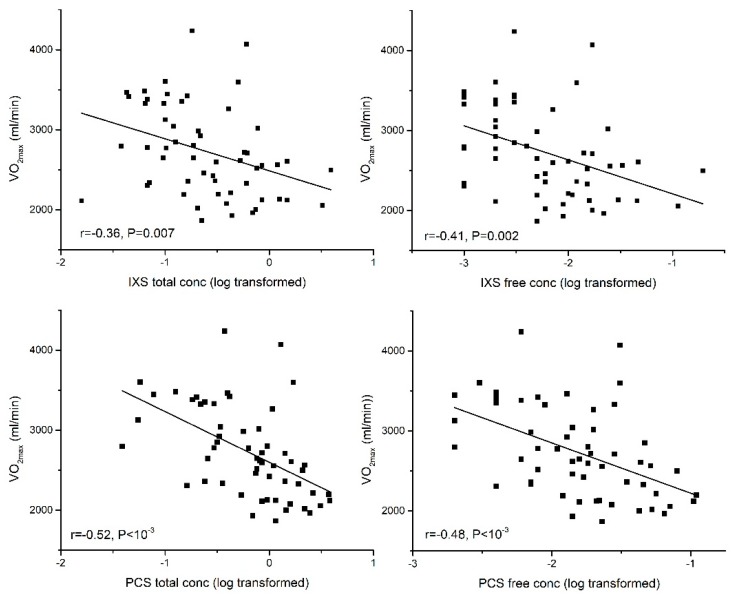
Association of serum total and free concentrations of indoxyl sulfate (IXS) and p-cresyl sulfate (PCS) with aerobic exercise capacity (VO_2max_).

**Figure 5:**
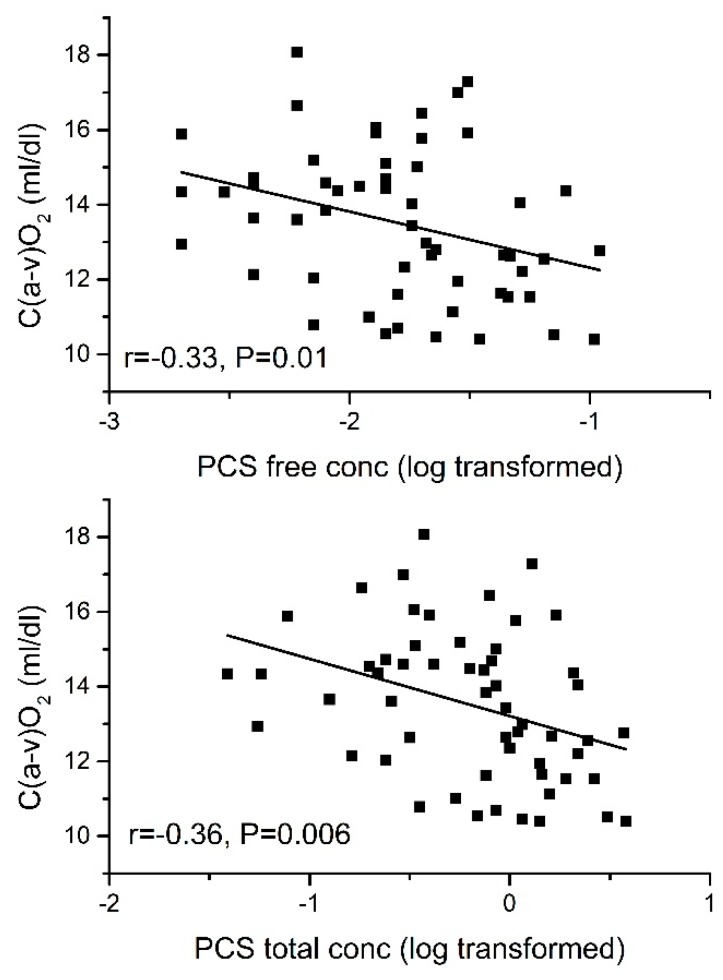
Association between serum total and free concentration of p-cresyl sulfate (PCS) and arterio-venous O_2_ difference [C(a-v)O_2_].

In partial least squares (PLS) multiple regression analysis, serum free concentrations of IXS and PCS were independently associated with percentage predicted CPO_max_ for age even after controlling for eGFR. The standardised coefficients (β) were −0.35 for IXS and −0.38 for PCS. An independent association was also demonstrated between free IXS and free PCS concentration and percentage of predicted VO_2max_ for age even after controlling for eGFR_._ The standardised coefficients (β) were −0.34 for IXS and −0.30 for PCS. Furthermore, an independent association was demonstrated between free PCS and C(a-v)O_2_ (β = −0.26). For these associations, all *p* are <0.05. The associations of IXS and PCS with CPO_max_ and VO_2max_ are shown in Figure 3 and Figure 4. Figure 5 shows the association between PCS and C(a-v)O_2_.

### 2.5. Association between Non-Protein-Bound Uremic Toxin and Peak Cardiac Performance and Aerobic Exercise Capacity

No correlation was seen between uric acid, a representative small molecule, and the above measures of cardiac performance or the measures of aerobic exercise capacity in the study cohort.

### 2.6. Association between PBUTs and Cardiac Dimensions

Left ventricular hypertrophy (LVH) (LVMI > 116 g/m^2^) was present in 14.6% of the patients, and LV concentric remodelling was present in 37.5% of the patients. No association between PBUTs and cardiac dimensions such as left ventricular mass, left ventricular mass index, relative wall thickness, left ventricular diameter in systole or diastole, or left atrial diameter was seen. However, interventricular septal thickness showed a positive correlation with the serum free concentrations of IXS (r = 0.33, *p =* 0.02) and PCS (r = 0.32, *p =* 0.03) (Table 4). The cardiac peak power-to-mass ratio expressed as peak CPO (in watts) per 100 g of left ventricular mass showed a significant negative correlation with the serum free concentrations of IXS (r = −0.29, *p =* 0.04), PCS (r = −0.29, *p =* 0.04), and PCG (r = −0.34, *p =* 0.02).

### 2.7. Effect of Beta Blockers

Of the 11 patients who were on beta blockers, 10 of them were in CKD stages 4 or 5. Therefore, linear regression was performed, controlling for eGFR, to compare haemodynamic parameters between CKD patients who were on beta blockers and those who were not. The results showed that there were no significant differences in CPO_max_ (Δ −0.04 W, *p =* 0.87) or peak CO (Δ 0.38 L/min, *p =* 0.63). The mean peak HR was lower in the beta blocker group by 28.15 beats/min (*p* < 10^−3^), whereas the mean peak SV was greater in the beta blocker group by 30.65 mL (*p* < 10^−3^), offsetting the reduction in peak HR.

## 3. Discussion

This study demonstrated for the first time the association between protein-bound uremic toxins and subclinical cardiac dysfunction in CKD. Of the 30 or more protein-bound uremic toxins that have previously been reported [1], we tested the leading candidates that might be cardiotoxic, and we found that both free IXS and free PCS were independently and significantly associated with subclinical cardiac dysfunction in CKD.

The present study is the first instance where the association between subclinical cardiac dysfunction, the precursor of symptomatic heart failure, and PBUTs has been shown. Although previous studies demonstrated an association between IXS and the incidence of HF in CKD [10], no association was demonstrable between IXS levels and markers of cardiac dysfunction such as LV ejection fraction or serum B-type natriuretic peptides (BNP) [11]. This is probably because the studies relied on resting measures or surrogate markers of cardiac dysfunction.

The resting performance of a failing myocardium is kept as normal as possible by compensatory mechanisms; hence, measurements of the resting performance of the heart are poorly discriminatory between healthy and diseased states [12]. In our study we therefore measured the peak performance of the heart, which exposed the subclinical cardiac dysfunction in CKD and demonstrated its relationship with PBUTs. In addition, the study also demonstrated an association between individual uremic toxins and haemodynamic characteristics such as peak cardiac output (peak Qt), peak mean arterial pressure (peak MAP), and peak heart rate (peak HR), which are markers of volume generating capacity, pressure generating capacity, and chronotropic reserve of the heart, respectively.

The study has also demonstrated for the first time the independent association between PBUTs and aerobic exercise capacity. Fick’s equation states that aerobic exercise capacity is a product of cardiac output and arteriovenous O_2_ difference [${\mathrm{VO}}_{2}=Q_{t}\times C\left(a-v\right)O_{2}$]. The study showed that the PBUTs were not only associated with impaired cardiac function but also with impaired arteriovenous O_2_ difference, which is a measure of peripheral O_2_ extraction by exercising skeletal muscles. IXS has been shown to accumulate in skeletal muscles in experimental uremia and induce metabolic alterations [13]. This could be a potential mechanism of skeletal myopathy related to PBUTs.

In the present study, in asymptomatic CKD patients, in the absence of co-morbid cardiac disease or diabetes, only 14.6% of the subjects had LVH; in addition, the study did not demonstrate any association between LVMI and PBUTs. However, there was association between PBUTs and early changes of cardiac hypertrophy such as interventricular septal thickness. More importantly, there was association between PBUTs and the LV power-to-mass ratio, a marker of cardiac pathological remodelling.

In the present study, beta blockade therapy was not shown to influence cardiac power. The reduction in peak HR associated with beta blockade was offset by the increase in SV; hence, there was no net effect on cardiac performance. Almost all CKD patients were treated with RAAS blockade, but if this has affected the results, the effect was uniform.

Mechanistic insights into the cardiotoxicity of PBUTs have been gained predominantly through the investigation of biochemical pathways using cultured cardiomyocytes or fibroblasts in the presence of PBUTs [14]. IXS and PCS have been the most studied. Studies testing other toxins and in vivo studies are rare. Nevertheless, these studies offer some significant insights into the cardiotoxicity of PBUTs. IXS was shown to induce cardiomyocyte hypertrophy through activation of the NFkB pathway and induce fibrosis through inhibition of the AMPK pathway [15,16]. Furthermore, molecular signatures of pathological remodelling, such as increased expression of mRNA for beta MHC and ANP, were seen in IXS-induced cardiotoxicity [17]. In addition, increased expression of TGF beta and IL-6, which are potent mediators of cardiac fibrosis, has also been shown [17]. Induction of oxidative stress by PBUTs is also a recurrent finding. The mechanism is implicated in the profibrotic effect of IXS and the pro-apoptotic effect of PCS [18,19]. 

It is pertinent to note that these pathways involved in the cardiotoxicity of PBUTs have striking similarities to the pathways seen in the pathological remodelling of the heart that leads to heart failure [20]. Several decades of heart failure research have shown that the consequences of such pathological remodelling are impaired cardiomyocyte energetics, altered expression and function of contractile proteins, impaired excitation–contraction coupling, etc., leading to impaired cardiomyocyte function and eventually myocardial dysfunction [21].

Although the current literature demonstrates the role of PBUTs in inducing cardiac ultrastructural changes such as fibrosis and hypertrophy, studies on the effects of these toxins on the cardiomyocyte function are still lacking. We believe that the relationship shown in the present study between individual PBUTs and cardiac dysfunction and the biomechanical characteristics of such dysfunction would help in the selection of candidate toxins for further in vitro and in vivo studies wherein the effects of PBUTs on cardiomyocyte physiology could be evaluated. Thus, the present work could lead to several “reverse translational” studies to further the knowledge on the cardiotoxicity of uremic toxins. Ultimately, these may reveal novel therapeutic targets.

## 4. Limitations

The cross-sectional nature of the study only allows for the demonstration of an association between uremic toxins and cardiac dysfunction. Further longitudinal studies of uremic-toxin-reducing strategies are required to demonstrate a causal relationship between uremic toxins and cardiac dysfunction.

## 5. Conclusions

This study demonstrated the association between PBUTs and early subclinical cardiac dysfunction in CKD. Although the effect of PBUTs on cardiac structure and ultrastructure has been well studied in the past, the present study for the first time has highlighted the role of PBUTs in cardiac dysfunction. We believe that the study results will open new avenues of research in exploring the mechanisms of myocardial dysfunction induced by protein-bound uremic toxins.

## 6. Materials and Methods

### 6.1. Study Subjects

Asymptomatic male CKD patients (stages 2 to 5 (pre-dialysis), *n* = 56) were recruited from the renal outpatient clinic of a tertiary U.K. referral center for cardiopulmonary exercise testing (CPX). The inclusion criteria were age of >18 years, male gender, and CKD stage 2 to 5 (pre-dialysis). The exclusion criteria were diabetes mellitus; any known cardiac diseases (ischaemic, arrhythmic, or valvular); limitation of exercise ability due to significant musculoskeletal, cardiovascular, pulmonary, hepatic, neurological, or other nonrenal medical disorders; and clinical hypervolemia. The present study is limited to the male gender alone to minimise confounders that arise because of gender and body composition on central haemodynamics [22,23,24]. Venous blood samples were taken prior to CPX to assay uremic toxins. Routine renal biochemistry tests were also performed. The estimated glomerular filtration rate (eGFR) was calculated using the 4-variable modification of diet in the renal disease Modification of Diet in Renal Disease (MDRD) formula [25]. The study was approved by the South Yorkshire Research Ethics Committee [Ref: 11/H1310/8, Date: 1 November 2011]. All subjects provided informed written consent before participation. These clinical investigations conformed with the Declaration of Helsinki.

### 6.2. Assessment of Peak Cardiac Performance

A specialised cardiopulmonary exercise test (CPX) was employed to measure peak cardiac power (CPO_max_) and cardiac haemodynamics noninvasively using the CO_2_ rebreathing method. Full methodological details have been described in previous reports [9,26,27]. A summary of the methodology is presented here.

#### Cardiopulmonary Exercise Test

Resting measures: The subjects had resting measurements taken for O_2_ consumption and CO_2_ production, respiratory rate, and cardiac output using a Medgraphics CardiO_2_ Analytic System (Medgraphics Corp., St. Paul, MN, USA). Resting cardiac output was calculated using the Collier CO_2_ rebreathing method [28,29].

Determination of aerobic capacity (VO_2max_): Subjects then underwent an incremental exercise test on a treadmill according to a standard Bruce protocol, and every 3 min, the speed and incline of the treadmill were increased according to the protocol until the subjects reached volitional exhaustion. Throughout the treadmill test, O_2_ consumption, CO_2_ production, end-tidal partial pressure of CO_2_, tidal ventilation, and respiratory rate were measured using breath-to-breath analysis. The ventilatory (“anaerobic”) threshold was measured by the V-slope method [30]. A 12-lead ECG was monitored throughout, and the subject’s heart rate (HR) was obtained from this. Blood pressure (BP) was measured at rest and at 3 min intervals up to, and including, maximal exercise by auscultation and sphygmomanometry.

Determination of cardiac output: After resting at least 40 min, a second treadmill test was performed. The speed and incline of the treadmill were adjusted manually. The subjects exercised on the treadmill to 95% of their VO_2 max_ as established in the incremental exercise test. Two or three cardiac output measurements were made using the Defare’s CO_2_ rebreathing method [31]. The blood pressure was measured using a sphygmomanometer after each determination of cardiac output. Peak cardiac power output is calculated using the formula $CPO=\left(Q_{t}\times MAP\right)\times K$ where Q_t_ is the cardiac output, MAP is the mean arterial pressure, and *K* (2.22 × 10^−3^) is a conversion factor to convert CPO into the SI unit of power, Watt. MAP is calculated using the formula MAP=DBP+0.412 (SBP−DBP) where SBP and DBP are systolic and diastolic blood pressures, respectively. VO_2_ is calculated using Fick’s equation, $VO_{2}=Q_{t}\times C\left(a-v\right)O_{2}$, where C(a-v)O_2_ is the arteriovenous O_2_ difference which is a measure of peripheral O_2_ extraction by the exercising skeletal muscles. Simultaneous measurement of Q_t_ and VO_2_ in the present study enabled the estimation of C(a-v)O_2_ using the above equation.

### 6.3. Echocardiogram

A 2-dimensional study was performed in the standard parasternal long-axis and short-axis planes followed by apical 4-chamber, apical 5-chamber, and apical 2-chamber planes to evaluate the cardiac structure and left ventricular contractility. The left ventricular ejection fraction (LVEF) was calculated by Simpson’s biplane method [32] and the left ventricular mass index (LVMI) was calculated by the Devereaux method [33]. The echocardiographic parameters were also utilised to measure the left ventricular power-to-mass ratio, a marker of pathological remodelling, by expressing peak cardiac power per 100 g of left ventricular mass (W/100 g of LV mass) [34].

### 6.4. Protein-Bound Uremic Toxin Assays

Blood samples were obtained from study subjects prior to CPX exercise testing. After clotting for 30 min the samples were centrifuged (10 min), and the serum stored as 0.5 mL aliquots in a −80 °C freezer. Indoxyl sulfate (IXS), p-cresyl sulfate (PCS), p-cresyl glucuronide (PCG), indole acetic acid (IAA), hippuric acid (HA), and 3-carboxy-4-methyl-5-propyl-2-furanpropionic acid (CMPF) were analysed by high-performance liquid chromatography (HPLC) at the nephrology department of the Ghent University Hospital, Ghent, Belgium as described in the previously published methodological papers [35,36].

For quantification of the total fraction, serum samples were deproteinised by heat (95 °C, 30 min). After cooling (10 min on ice), samples were filtered through a Centrifree-filter (cut-off: 30 kDa; Millipore, Billerica, MA, USA). To determine the free fraction, untreated serum samples were ultrafiltered prior to heating. Indoxyl sulfate (λex: 272 nm, λem: 374 nm), p-cresyl sulfate and p-cresyl glucuronide (λex: 264 nm, λem: 290 nm), and indole-3-acetic acid (λex: 272 nm, λem: 340 nm) were detected using a Waters 2475 fluorescence detector, while uric acid, hippuric acid and CMPF were detected using a Waters 996 photodiode array detector at 300 nm, 245 nm, and 254 nm respectively (Waters Alliance 2695 device (Waters, Zellik, Belgium) connected to a Waters fluorescence detector and a UV detector).

### 6.5. Statistical Analysis

CPO_max_ was the primary outcome measure. The association between CPO_max_ and serum total and free concentrations of PBUTs was evaluated using Pearson’s correlation. Logarithmic transformed values of PBUT concentration were used for analysis. Similar associations with other haemodynamic parameters such as peak cardiac output (Q_t_), peak mean arterial pressure (MAP) and peak heart rate (HR), and aerobic exercise capacity (VO_2max_) and peripheral O_2_ extraction (C(a-v)O_2_) were also assessed. Furthermore, the correlation between PBUTs and measures of cardiac dimension and LV power-to-mass ratio was also assessed.

The independent association between PBUTs and CPO_max_, controlling for eGFR, was assessed using partial least squares multiple regression analysis (PLS), as the use of a standard multiple regression analysis was precluded by collinearity between the independent variables. Absolute values of PBUT concentration (rather than log-transformed values) were used for such analysis. Free concentrations of PBUTs were used for analysis as only the free unbound fractions of the toxins are biologically active and, hence, clinically relevant. As CPO_max_ is influenced by age, percentage predicted CPO_max_ for age, as calculated using a previously published regression equation [22], was used as the dependent variable. Thus, in the PLS analysis, percentage predicted CPO_max_ was the dependent variable, and IXS, PCS, PCG, IAA, HA, and eGFR were the independent variables. Similar analyses were performed with percentage predicted VO_2max_ as the dependent variable. Data are presented as mean ± SD or median (25th pct, 75th pct) as appropriate. *p* < 0.05 is considered significant.

## Figures and Tables

**Table 1:** Patient characteristics.

Patient Characteristics	CKD 2 (*n* = 14)	CKD 3 (*n* = 15)	CKD 4 (*n* = 16)	CKD 5 (*n* = 11)	ANOVA (*p*)
Age (years)	38.1 ± 8.8	52.2 ± 9.1	48.6 ± 12.7	47.9 ± 15.4	0.01 *
BMI (kg/m^2^)	26.6 ± 3.6	28.6 ± 4.1	26.6 ± 4.2	29.5 ± 4.1	0.16
**Biochemistry**					
eGFR (mL/min)	73.5 ± 7.9	43.2 ± 7.9	21.5 ± 4.3	11.9 ± 2.4	<10^−3^ *
Creatinine (μmoL/L)	104.6 ± 10.9	158.1 ± 2.6	294.8 ± 56.7	550.1 ± 227.4	<10^−3^ *
Urea (mmoL/L)	7.3 ± 1.8	11.5 ± 2.4	20.1 ± 3.6	27.0 ± 8.7	<10^−3^ *
Calcium (mmoL/L)	2.4 ± 0.1	2.4 ± 0.1	2.3 ± 0.1	2.3 ± 0.2	0.54
Phosphate (mmoL/L)	1.1 ± 0.2	1.1 ± 0.1	1.2 ± 0.1	1.6 ± 0.7	0.001 *
Bicarbonate (mmoL/L)	28 ± 2.5	25.9 ± 2.5	22.9 ± 3.3	21.0 ± 3.5	<10^−3^ *
Uric acid (mg/dL)	8.6 ± 2.0	10.9 ± 2.3	9.7 ± 2.2	10.4 ± 2.2	0.05
PTH (pmoL/L)	6.1 ± 4.8	16.9 ± 25.7	25.4 ± 17.4	47.6 ± 38.9	0.005 *
Haemoglobin (g/dL)	15.1 ± 1.2	14 ± 1.4	13.2 ± 1.6	12.1 ± 1.3	<10^−3^ *
**CPX parameters**					
Peak Qt (L/min)	22.1 ± 2.1	20.1 ± 2.1	19.1 ± 2.2	19.1 ± 1.9	0.001 *
Peak MAP (mmHg)	106.4 ± 8.9	107.6 ± 7.9	104.2 ± 10.0	101.0 ± 8.9	0.32
Peak HR (beats/min)	169.7 ± 18.1	151.8 ± 12.8	149.3 ± 23.7	145.1 ± 18.5	0.007 *
CPO_max_ (W)	5.23 ± 0.76	4.79 ± 0.61	4.44 ± 0.55	4.29 ± 0.63	0.002 *
VO_2max_ (L/min)	3.13 ± 0.57	2.70 ± 0.47	2.61 ± 0.63	2.43 ± 0.30	0.009 *
Peak C(a-v)O_2_ (dL/min)	14.2 ± 2.1	13.4 ± 2.0	13.5 ± 2.04	12.8 ± 1.7	0.40

BMI: body mass index, Q_t_: cardiac output, MAP: mean arterial pressure, HR: heart rate, CPO_max_: peak cardiac power, VO_2max_: peak O_2_ consumption, C(a-v)O_2_: arteriovenous difference in O_2_ concentration.* *p* < 0.05 on ANOVA across chronic kidney disease (CKD) stages.

**Table 2:** Total and free concentrations of the assayed protein-bound uremic toxins (PBUTs).

Uremic Toxins		Serum Concentration Median (25th pct, 75th pct) (*n* = 56)
**IXS** (mg/dL)	Total	0.222 (0.101, 0.598)
	Free	0.006 (0.002, 0.015)
**PCS** (mg/dL)	Total	0.782 (0.303, 1.418)
	Free	0.017 (0.007, 0.031)
**PCG** (mg/dL)	Total	0.013 (0.004, 0.035)
	Free	0.011 (0.002, 0.029)
**IAA** (mg/dL)	Total	0.057 (0.040, 0.101)
	Free	0.005 (0.003, 0.011)
**HA** (mg/dL)	Total	0.255 (0.119, 0.379)
	Free	0.084 (0.038, 0.137)
**CMPF** (mg/dL)	Total	0.071 (0.035, 0.156)

Indoxyl sulfate (IXS), p-cresyl sulfate (PCS), p-cresyl glucuronide (PCG), indole acetic acid (IAA), hippuric acid (HA), and 3-carboxy-4-methyl-5-propyl-2-furanpropionic acid (CMPF).

**Table 3:** Correlation coefficients (r) between serum *total* concentration of PBUTs and parameters of cardiac function, structure, and exercise capacity.

Study Parameters	IXS	PCS	PCG	IAA	CMPF	HA
**CPO_max_**	−0.44 **	−0.58 **	−0.52 **	−0.33 *		−0.35 **
**Peak Q_t_**	−0.41 **	−0.48 **	−0.45 **	−0.27 *		−0.42 **
**Peak MAP**		−0.35 **	−0.30 *			
**Peak HR**			−0.41 **	−0.27 *		−0.30 *
**VO_2max_**	−0.36 **	−0.53 **	−0.43 **	−0.37 **		−0.30 *
**Peak C(a-v)O_2_**		−0.36 **	−0.26 *	−0.31 *		
**LVMI**						
**IVSd**	0.30 *					

Heat map showing negative correlations in 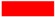 and positive correlations in 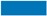. Blank spaces represent no significant correlation. ** *p* < 0.01 and * *p* < 0.05 on Pearson correlation. Log-transformed values of serum concentrations of uremic toxins were used for analysis. CPO_max_: peak cardiac power; peak Q_t_: peak cardiac output; MAP: mean arterial pressure; peak HR: peak heart rate; VO_2max_: aerobic exercise capacity; C(a-v)O_2_: arteriovenous O_2_ difference; LVMI: left ventricular mass index; IVSd: interventricular septal thickness at end diastole. Indoxyl sulfate (IXS), p-cresyl sulfate (PCS), p-cresyl glucuronide (PCG), indole acetic acid (IAA), hippuric acid, and 3-carboxy-4-methyl-5-propyl-2-furanpropionic acid (CMPF).

**Table 4:** Correlation coefficients (r) between serum *free* concentration of PBUTs and parameters of cardiac function, structure, and exercise capacity.

Study Parameters	IXS	PCS	PCG	IAA	HA
**CPO_max_**	−0.48 **	−0.51 **	−0.52 **	−0.40 **	−0.29 *
**Peak Q_t_**	−0.41 **	−0.44 **	−0.45 **	−0.33 *	−0.40 **
**Peak MAP**		−0.29 *	−0.29 *		
**Peak HR**			−0.41 **	−0.31 *	
**VO_2max_**	−0.41 **	−0.48 **	−0.42 **	−0.41 **	−0.38 **
**Peak C(a-v)O_2_**		−0.332 *		−0.31 *	
**LVMI**					
**IVSd**	0.33 *	0.32 *			

Heat map showing negative correlations in 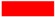 and positive correlations in 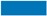. Blank spaces represent no significant correlation. ** *p* < 0.01 and * *p* < 0.05 on Pearson correlation. Log-transformed values of serum concentrations of uremic toxins were used for analysis. CPO_max_: peak cardiac power; peak Q_t_: peak cardiac output; MAP: mean arterial pressure; peak HR: peak heart rate; VO_2max_: aerobic exercise capacity; C(a-v)O_2_: arteriovenous O_2_ difference; LVMI: left ventricular mass index; IVSd: interventricular septal thickness at end diastole. Indoxyl sulfate (IXS), p-cresyl sulfate (PCS), p-cresyl glucuronide (PCG), indole acetic acid (IAA), and hippuric acid (HA).
